# Supplementary figures and images for: Modeling second-order boundary perception: A machine learning approach
Source: PLoS Comput Biol. 2019 Mar 18;15(3):e1006829. doi: 10.1371/journal.pcbi.1006829 (PMC6438569; doi:10.1371/journal.pcbi.1006829)

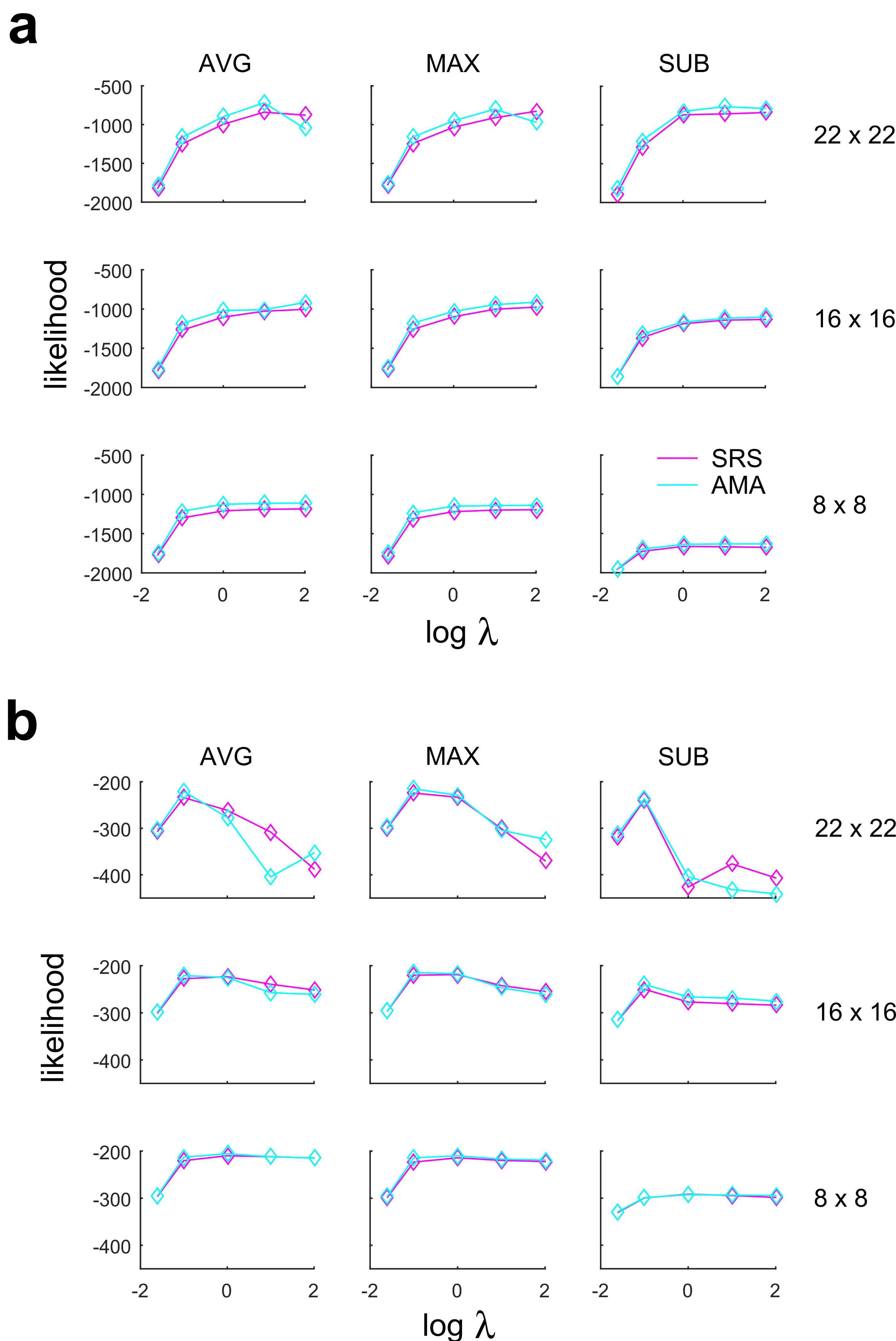

Supplement: S1 Fig — Optimization was performed using k-fold cross-validation (k = 4). (a) Likelihood of training set (averaged over folds) for various values of λ for different down-sampling sizes and pooling rules. (b) Likelihood of validation set (averaged over folds) for various values of λ. (TIF) [file pcbi.1006829.s001.tif]

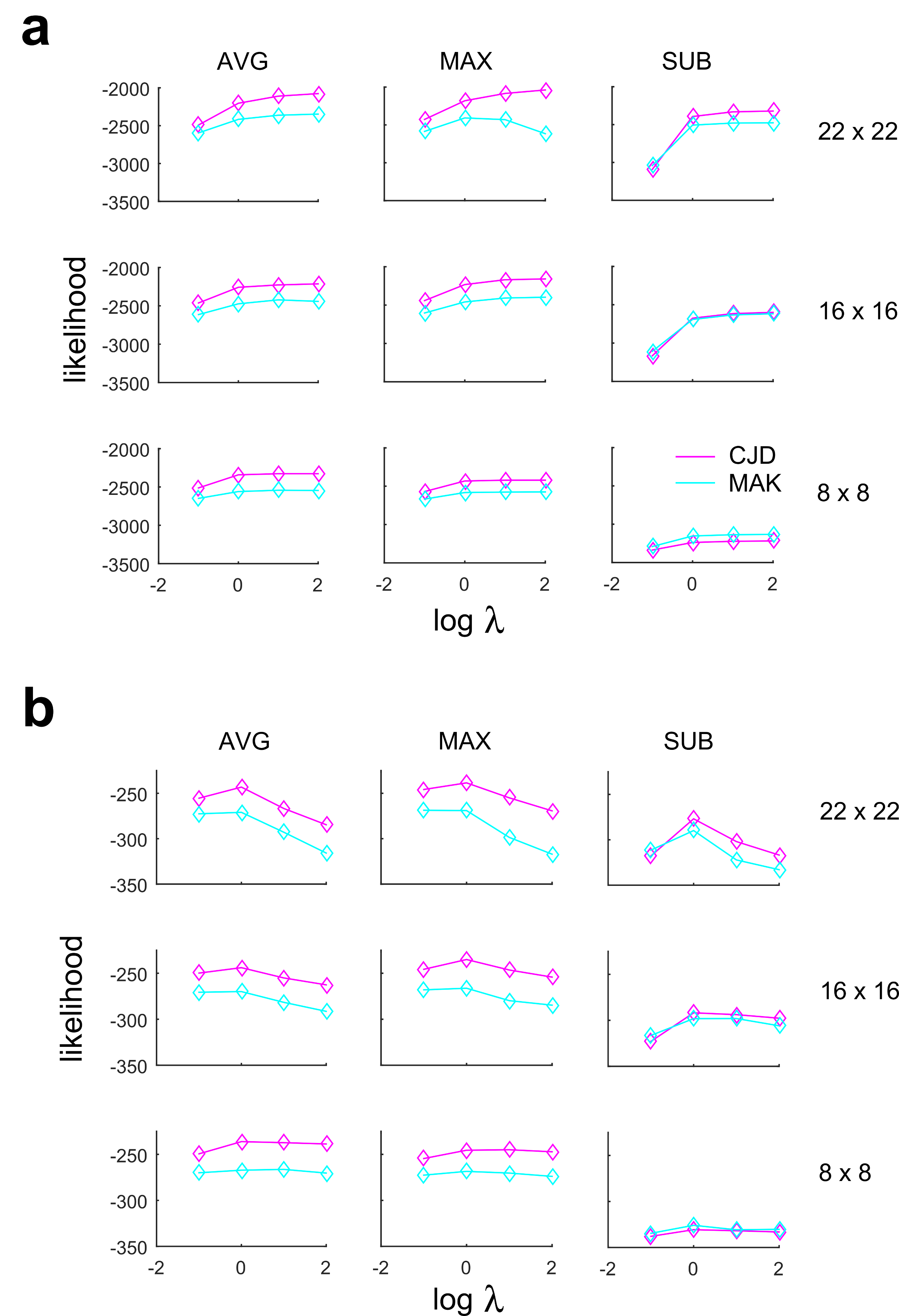

Supplement: S2 Fig — (TIF) [file pcbi.1006829.s002.tif]

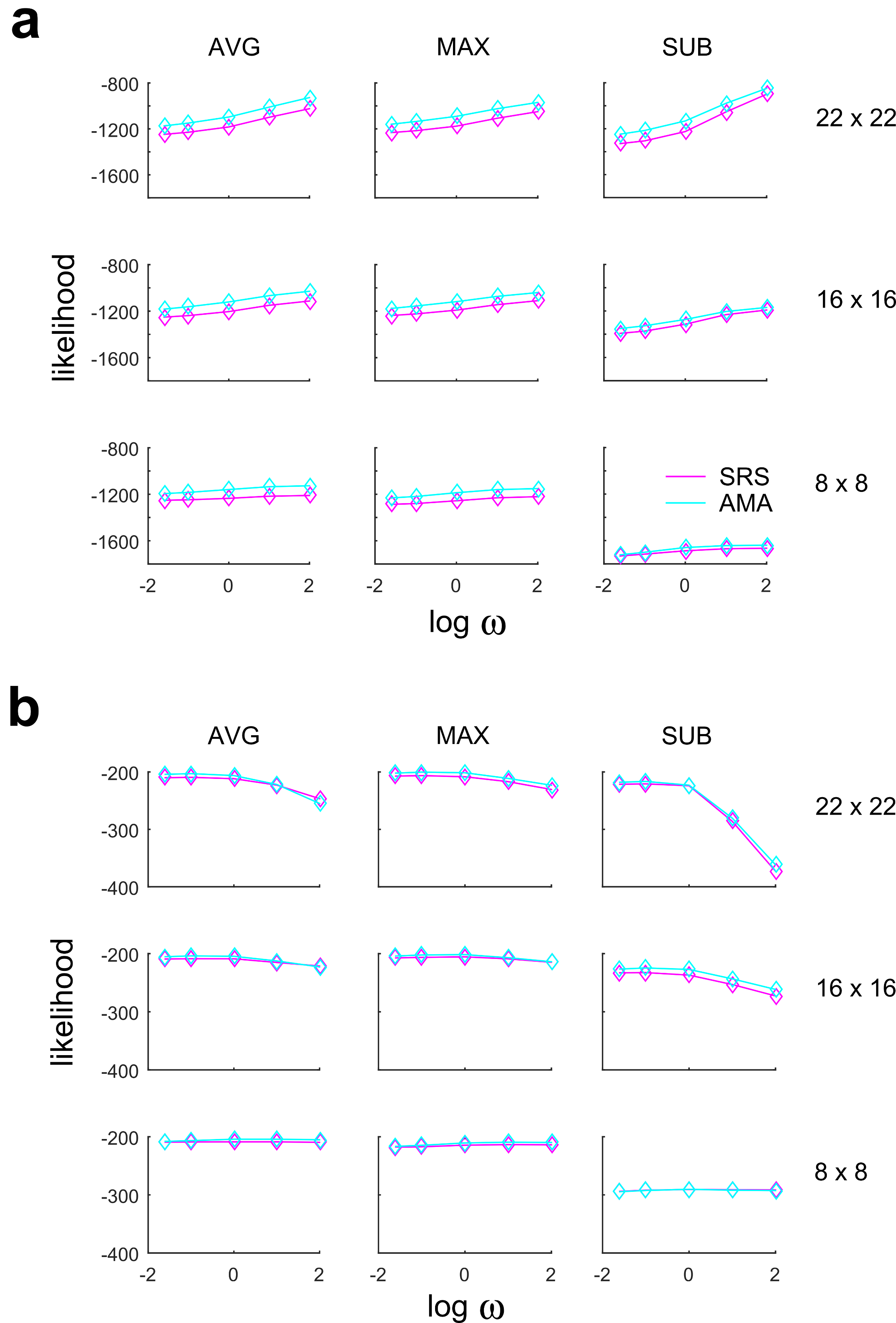

Supplement: S3 Fig — Optimization was performed using k-fold cross validation (k = 4). (a) Likelihood of training set (averaged over folds) for various values of ω for several down-sampling sizes and pooling rules. (b) Likelihood of validation set (averaged over folds) for various values of ω. (TIF) [file pcbi.1006829.s003.tif]

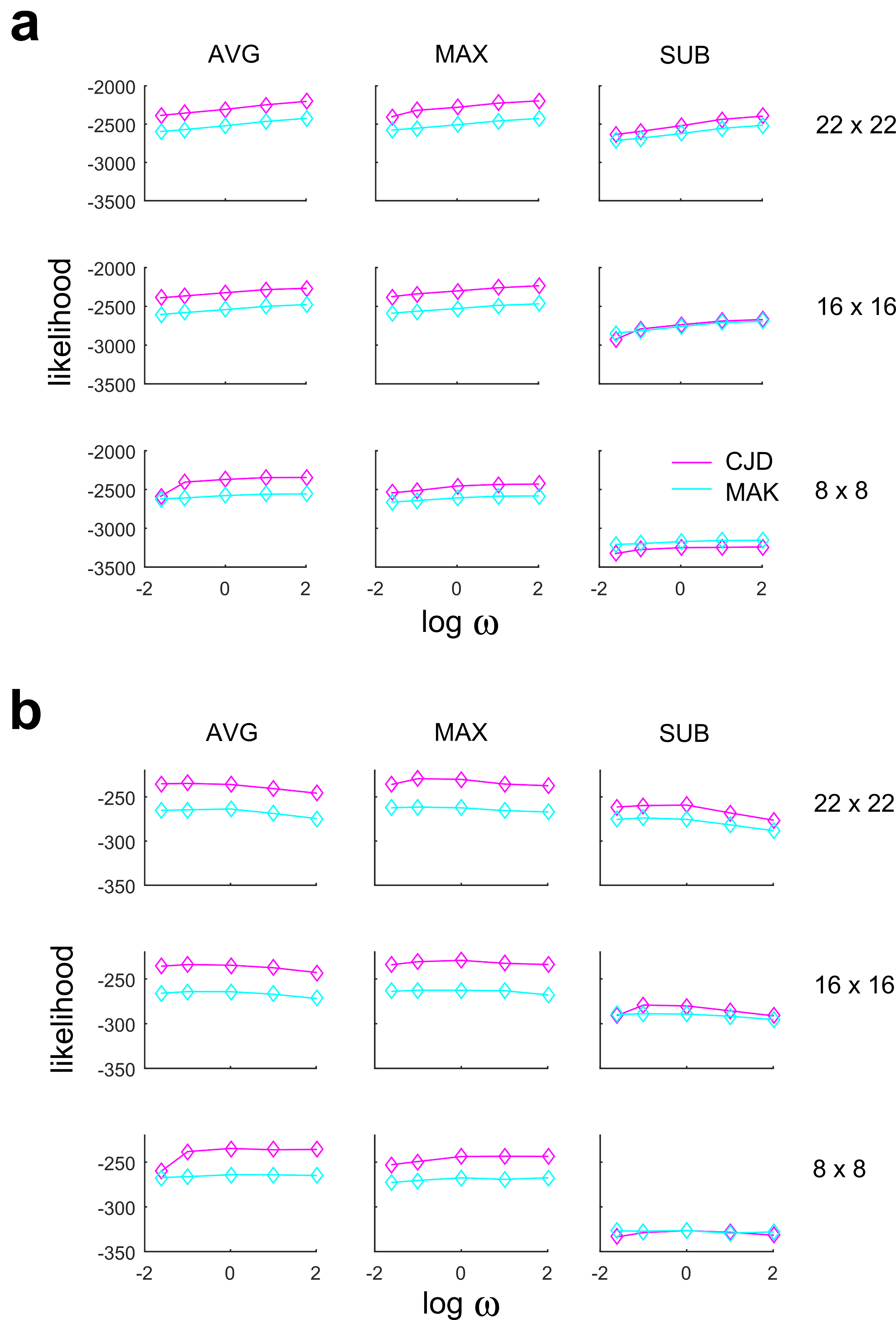

Supplement: S4 Fig — (TIF) [file pcbi.1006829.s004.tif]

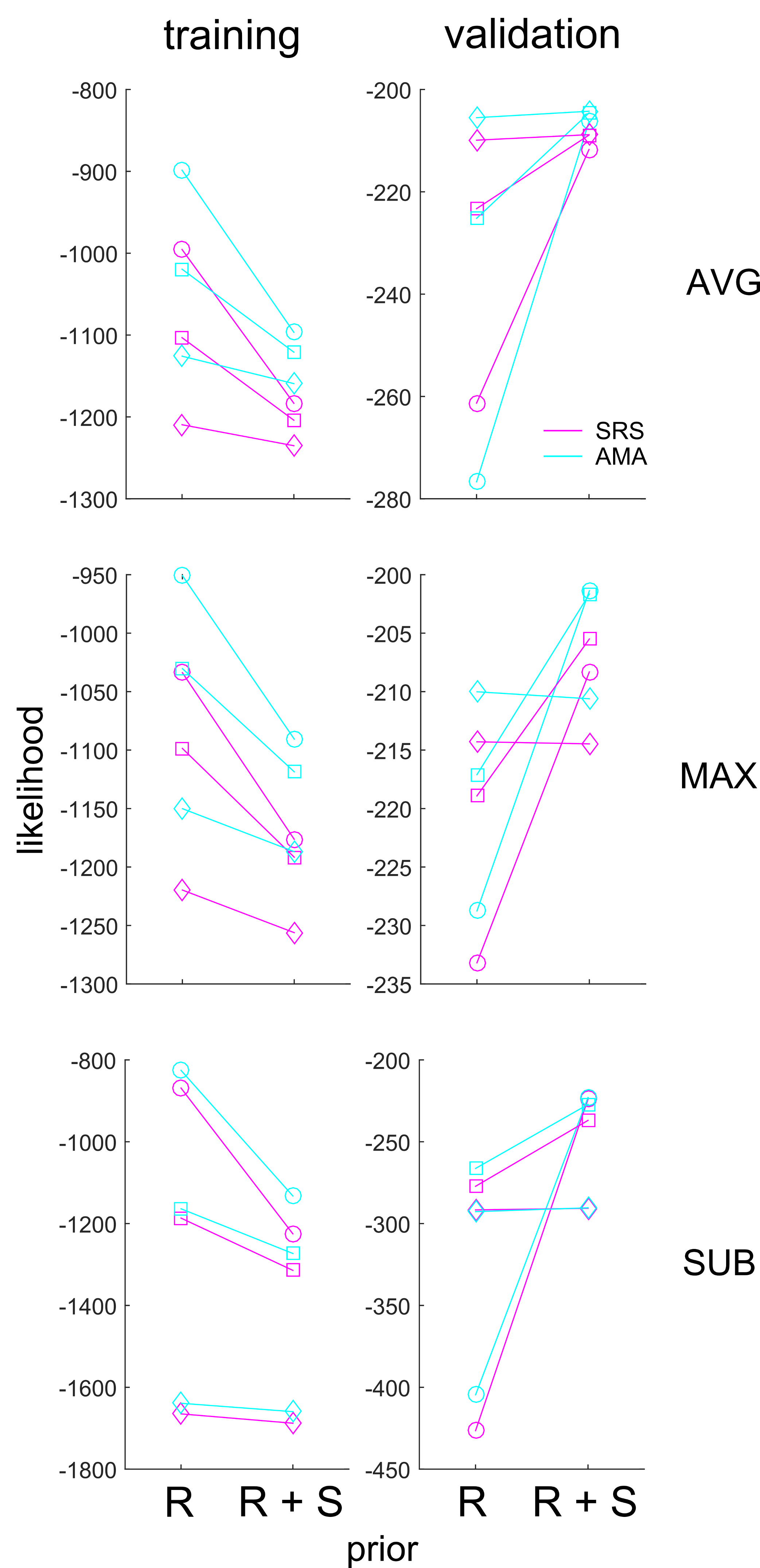

Supplement: S5 Fig — We see that for all three pooling rules (AVG, MAX, SUB), addition of the smoothing constraint decreases the fit to the training data (left), but improves generalization to the validation data (right). (TIF) [file pcbi.1006829.s005.tif]

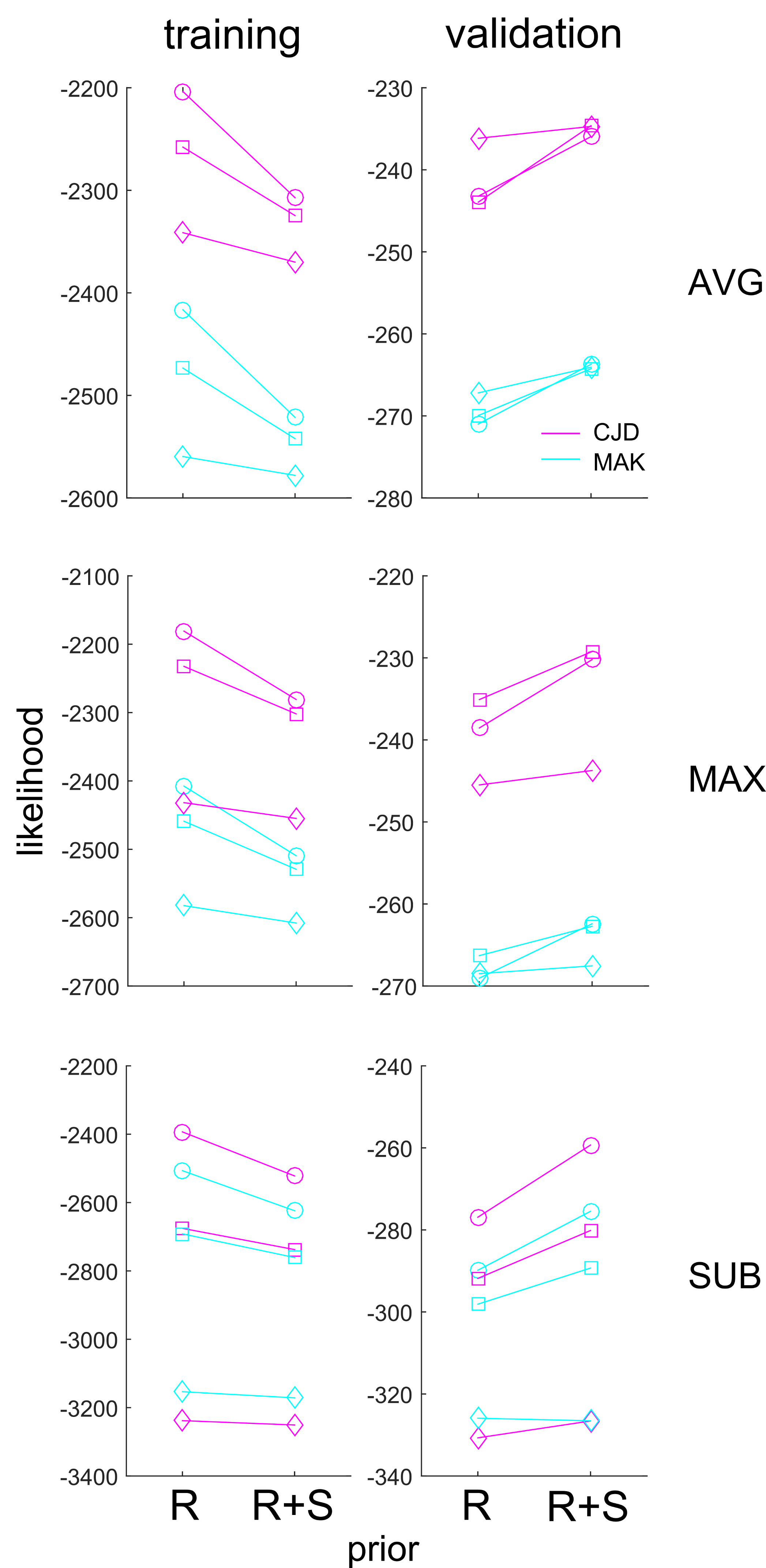

Supplement: S6 Fig — (TIF) [file pcbi.1006829.s006.tif]

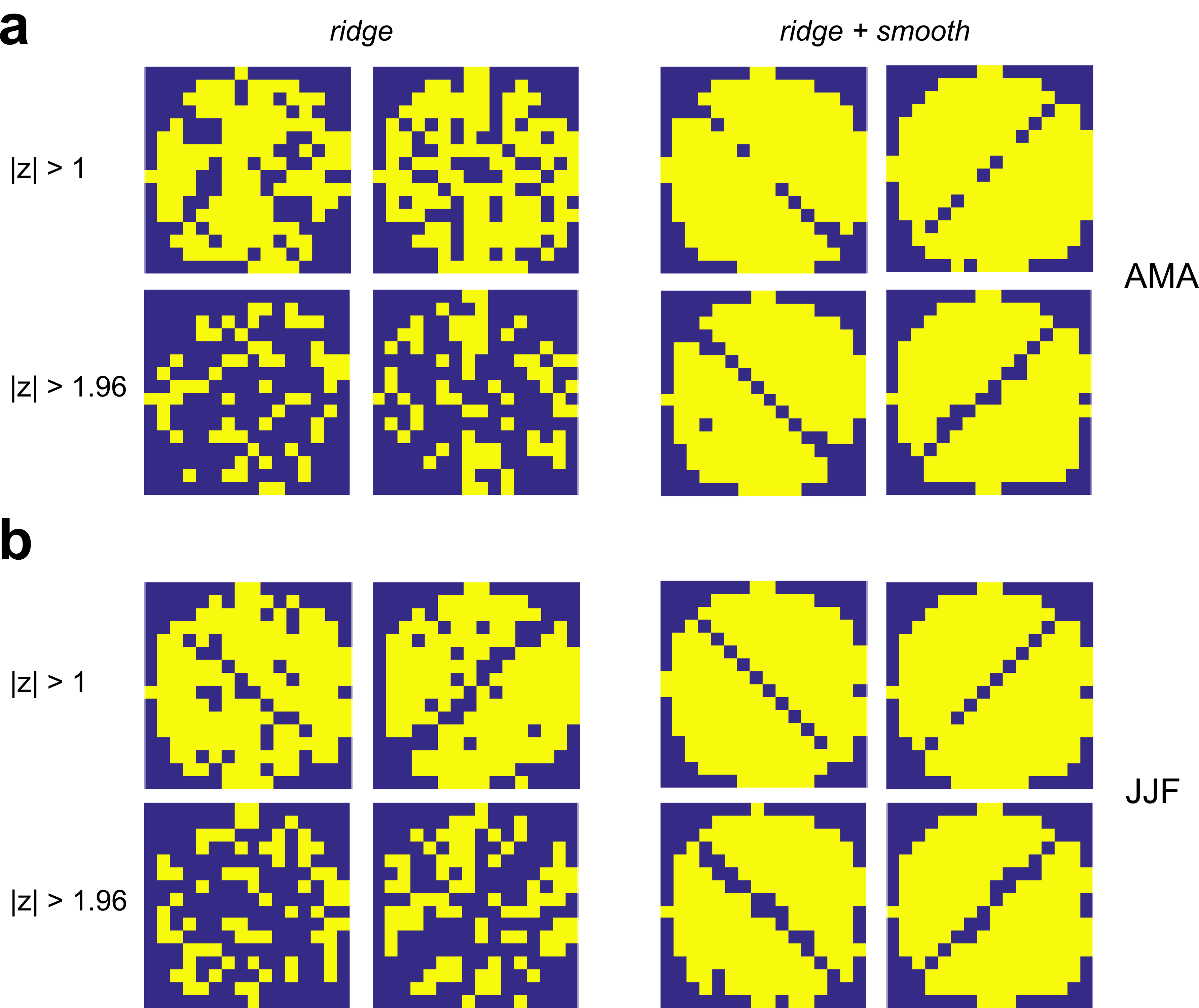

Supplement: S7 Fig — Left columns show ridge prior, right show ridge + smooth prior. (a) Observer AMA in Experiment 1-VAR. (b) Observer JJF in Experiment 1-FIX. (TIF) [file pcbi.1006829.s007.tif]

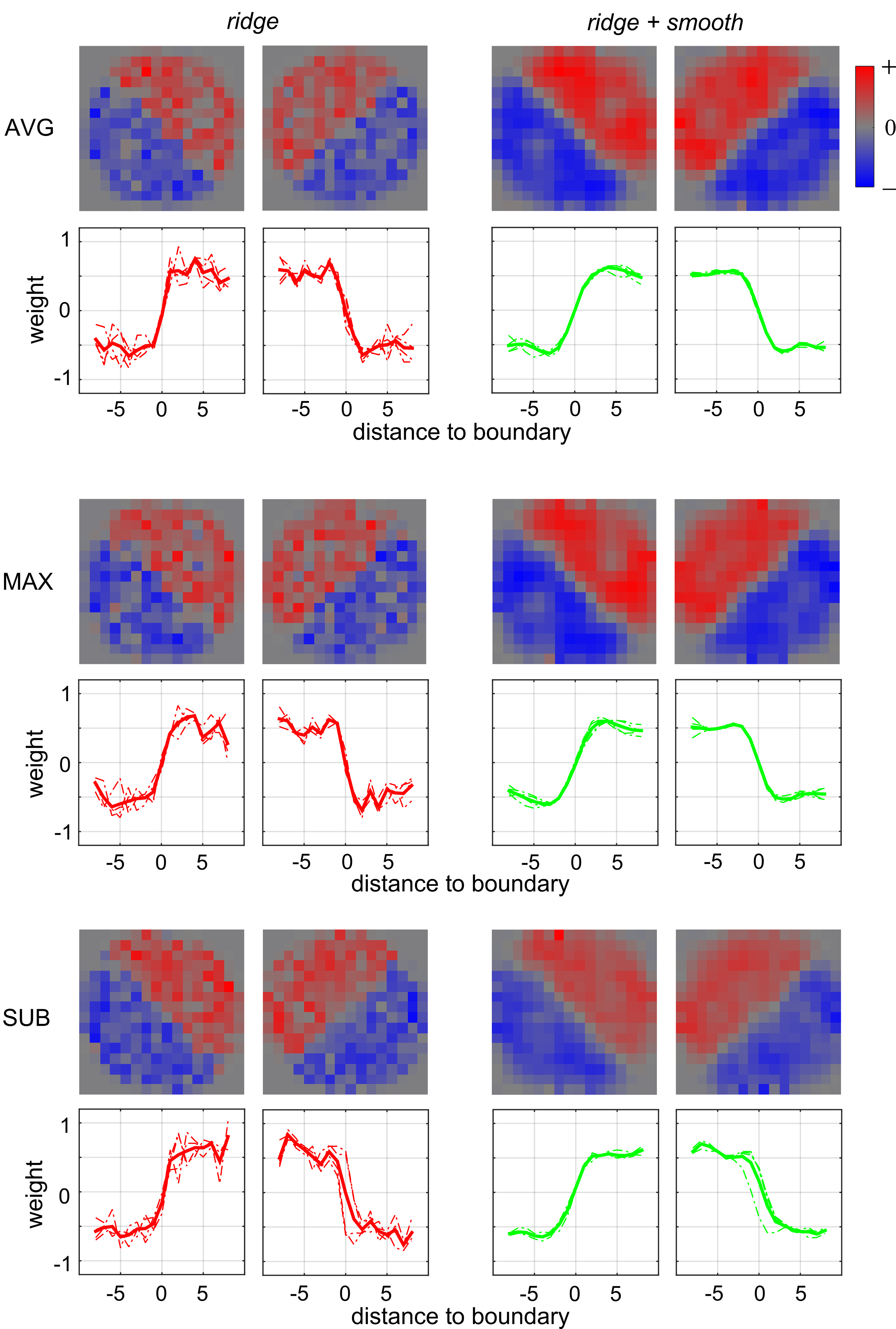

Supplement: S8 Fig — Organization is as in Fig 3, except that in the 1-D plots the thin dashed lines are fits to individual folds (k = 4), and the thick line is the average. (TIF) [file pcbi.1006829.s008.tif]

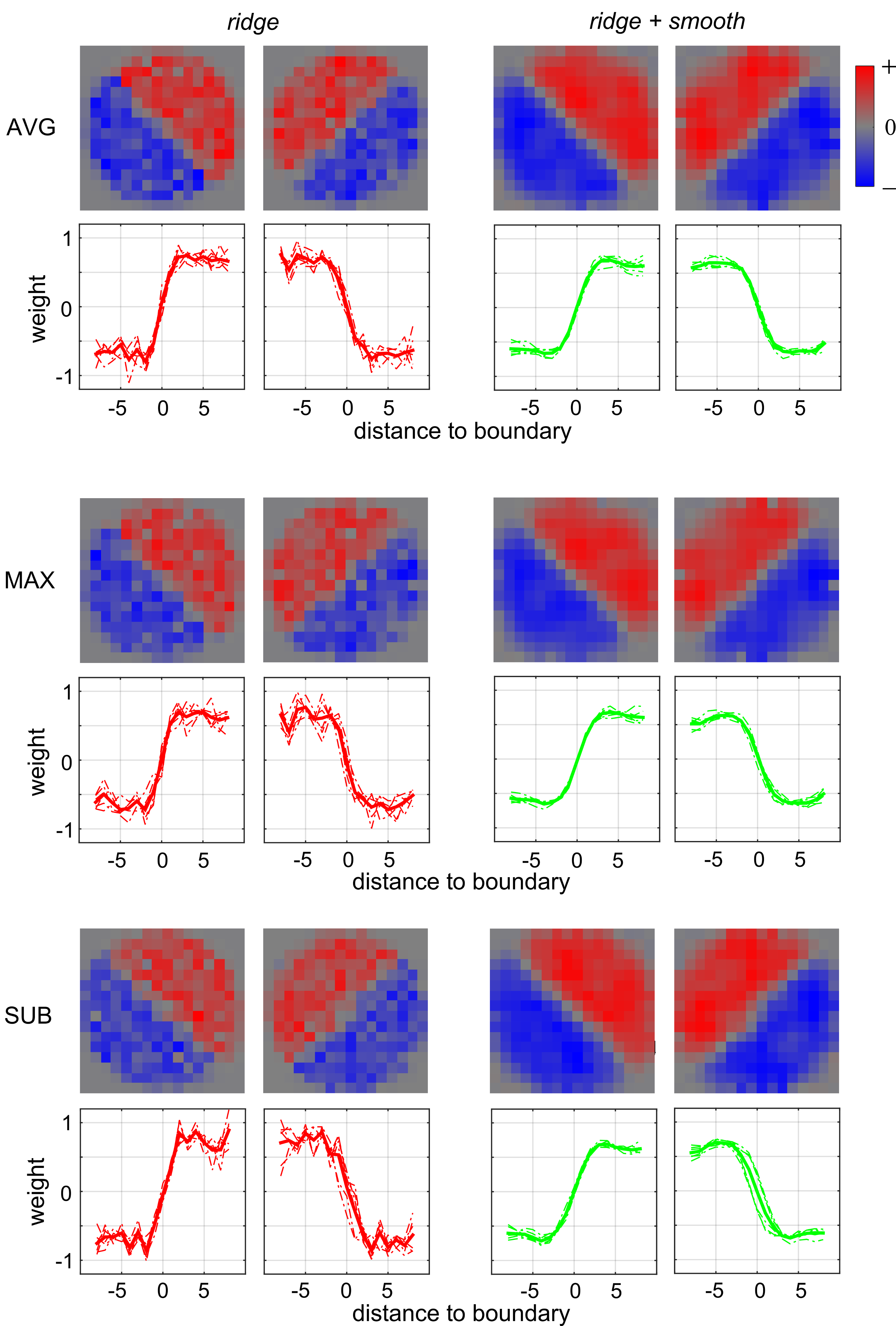

Supplement: S9 Fig — (TIF) [file pcbi.1006829.s009.tif]

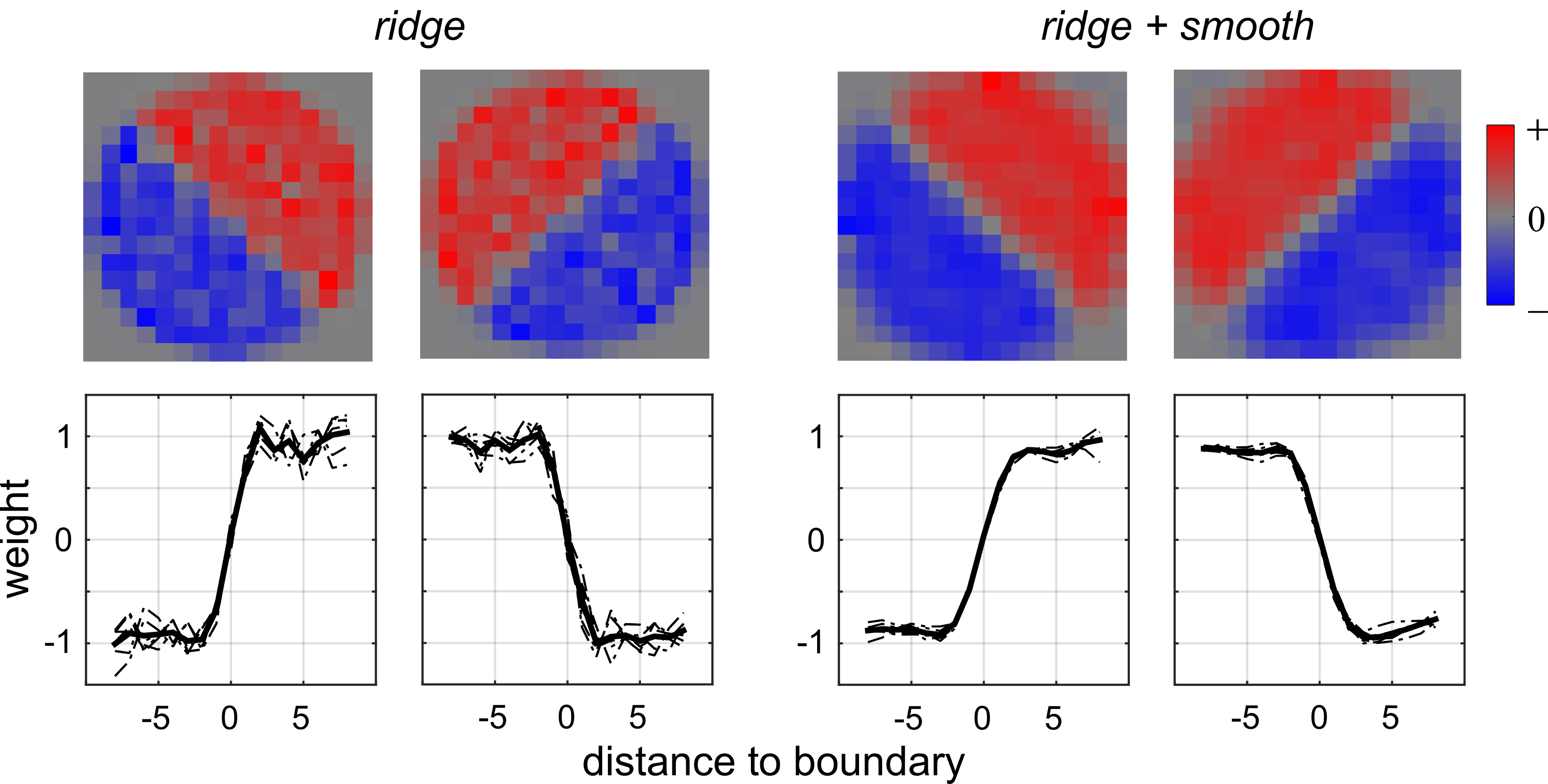

Supplement: S10 Fig — (TIF) [file pcbi.1006829.s010.tif]

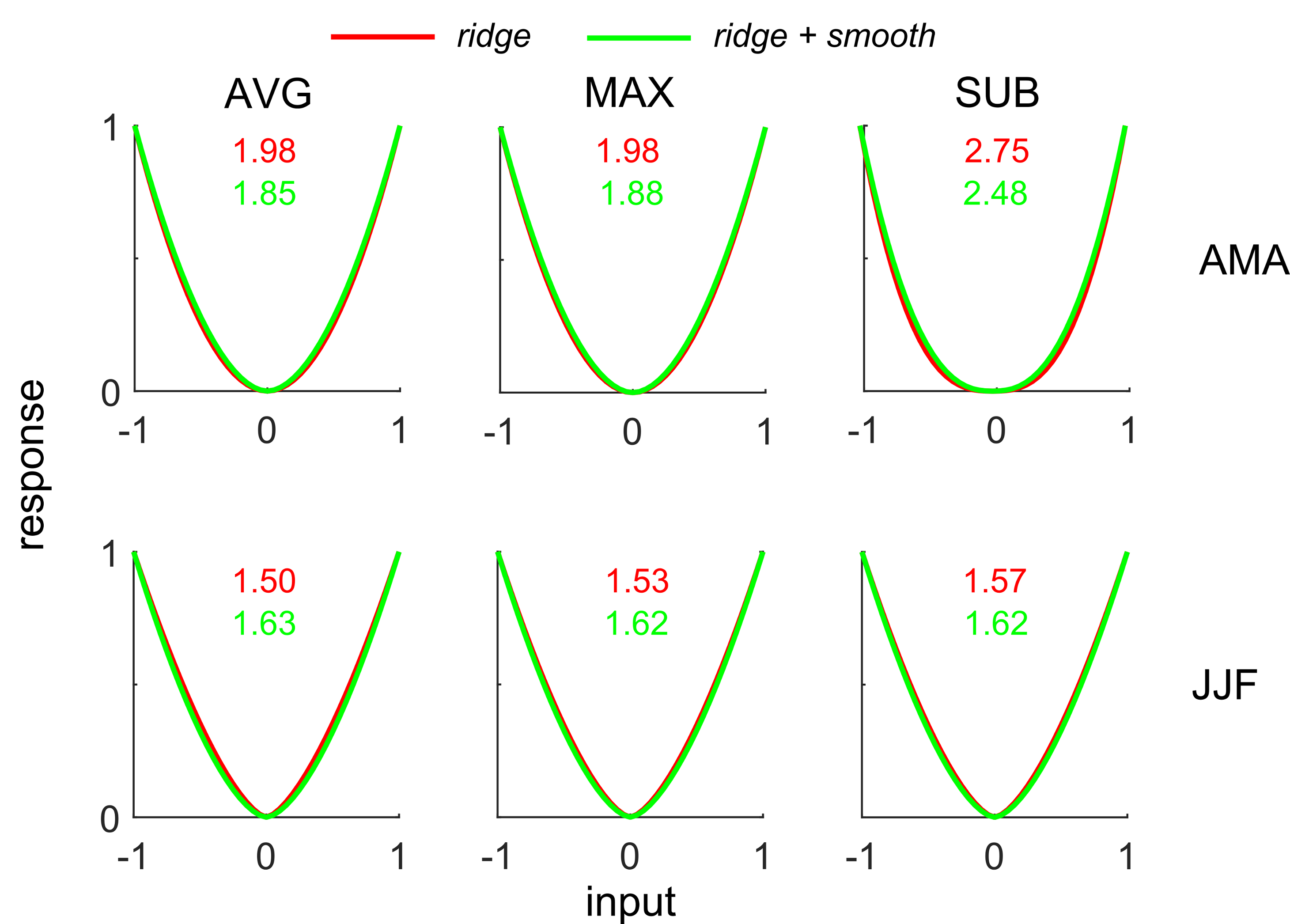

Supplement: S11 Fig — (TIF) [file pcbi.1006829.s011.tif]

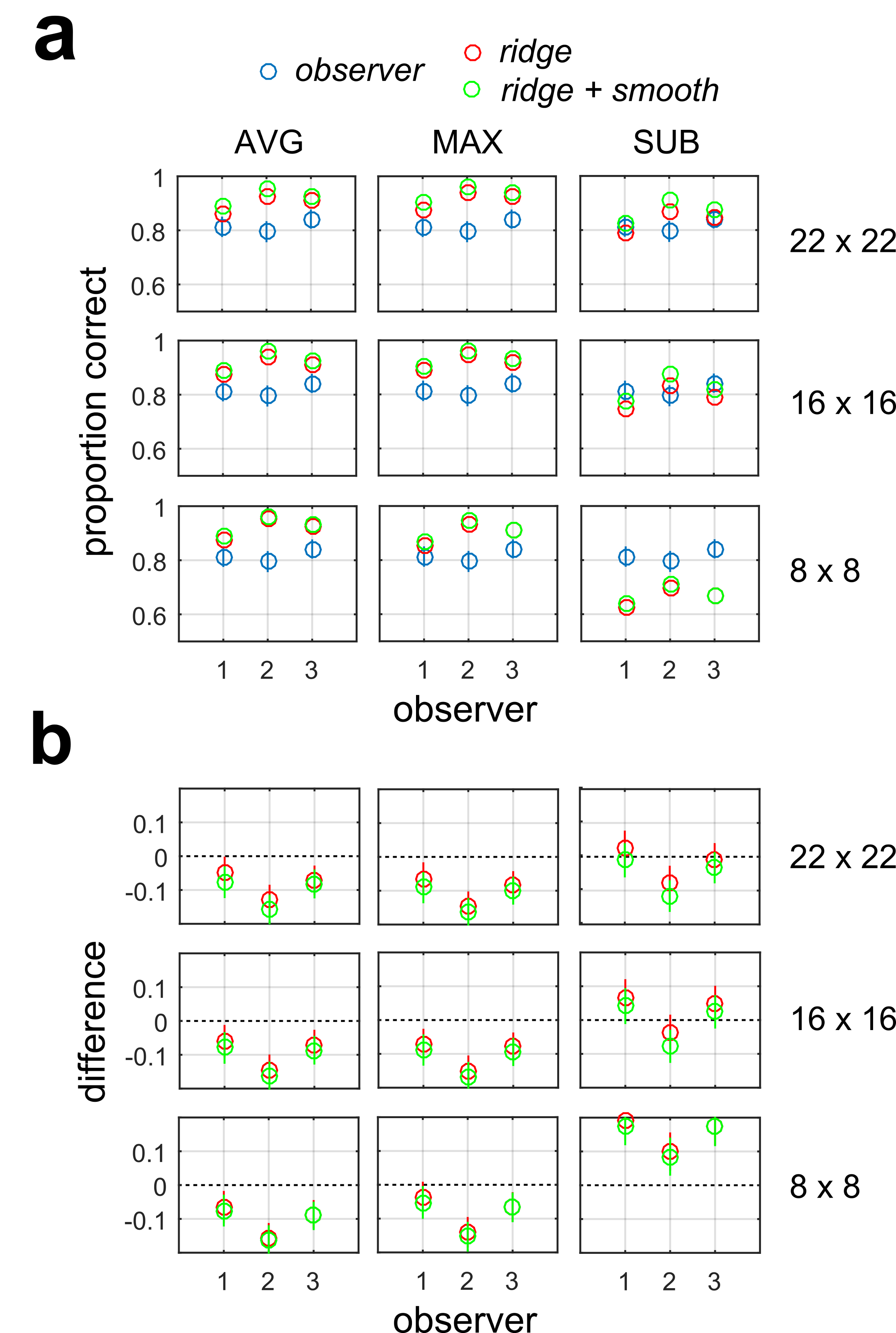

Supplement: S12 Fig — (a) Performance of observers (blue) and models with both priors. (b) Difference between observer and model performance. (TIF) [file pcbi.1006829.s012.tif]

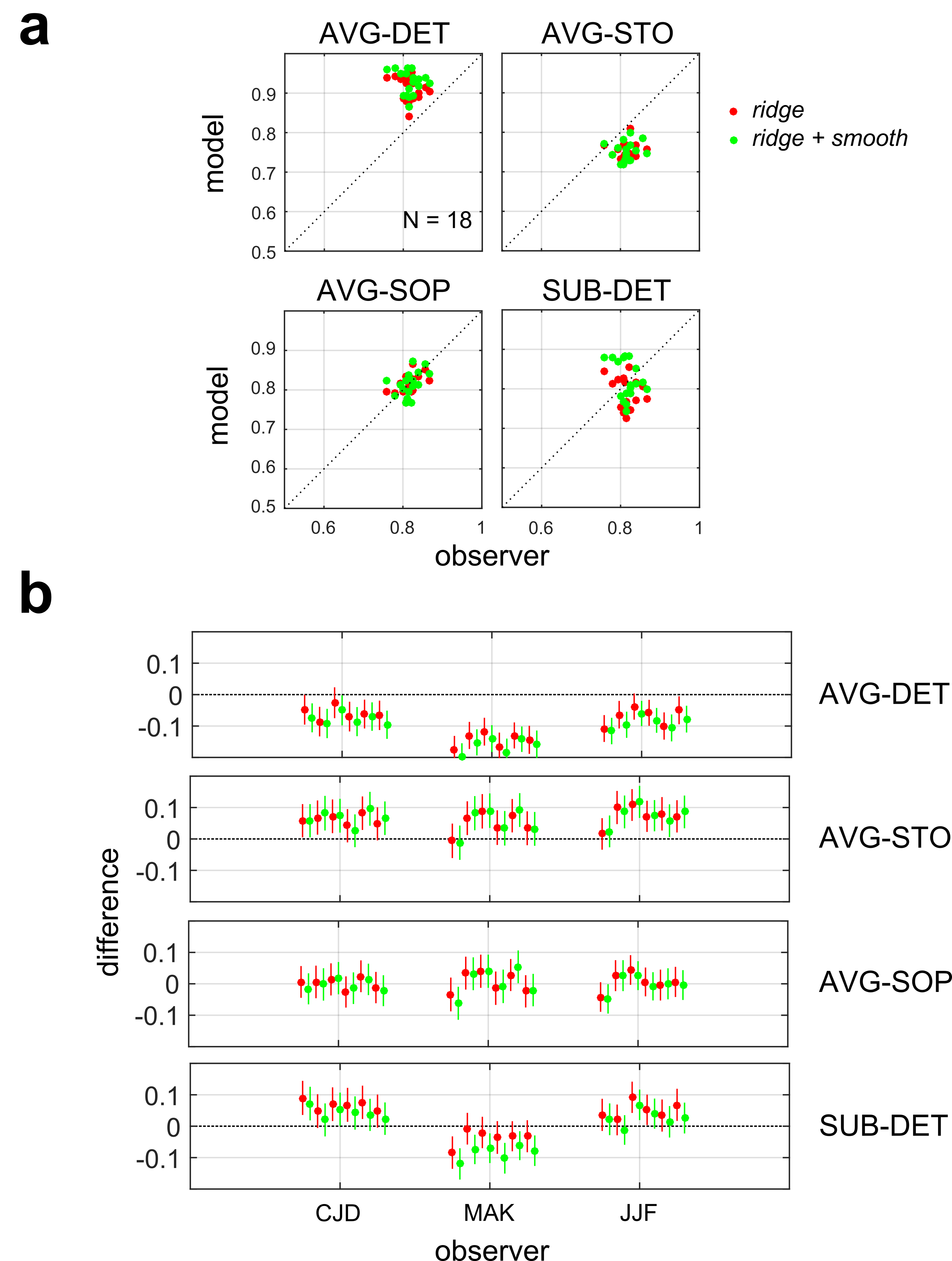

Supplement: S13 Fig — (a) Scatterplots of model vs. observer performance, averaged across observers, for all test folds (N = 18 folds, 6 per observer). We also show the stochastic AVG model with an internal noise parameter, β, optimized to fit observer performance (on the training set, which was not used for testing), denoted AVG-SOP, bottom left panel. Values of the noise parameter β for each observer are shown in S4 Table. (b) Difference between observer and model performance (observer—model) for each individual test fold (3 observers, 6 folds per observer) for all models shown in (a). Lines show 95% confidence intervals of the difference (binomial proportion difference test). (TIF) [file pcbi.1006829.s013.tif]

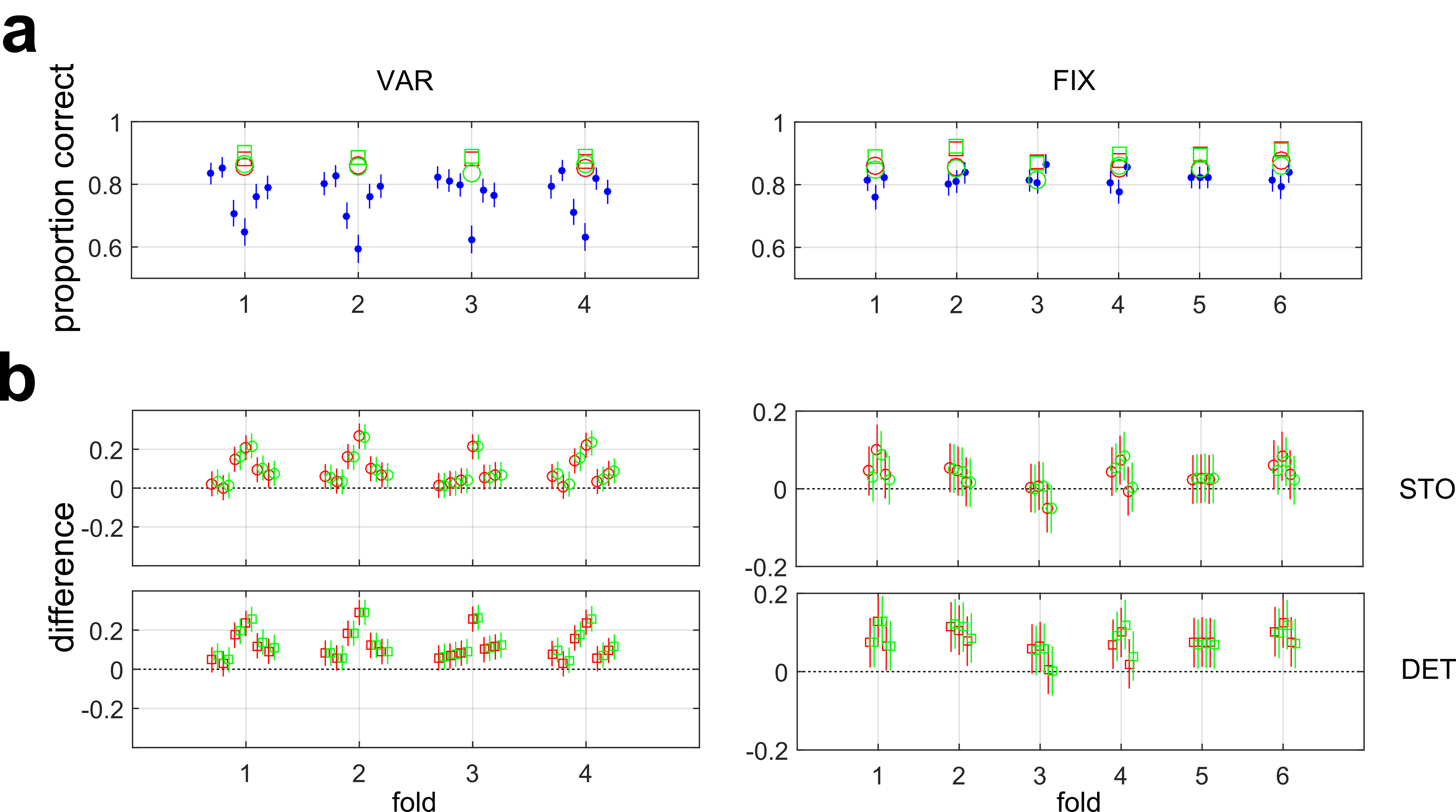

Supplement: S14 Fig — (a) Proportion correct for models and observers (blue dots). (b) Difference between observer and model performance. (TIF) [file pcbi.1006829.s014.tif]

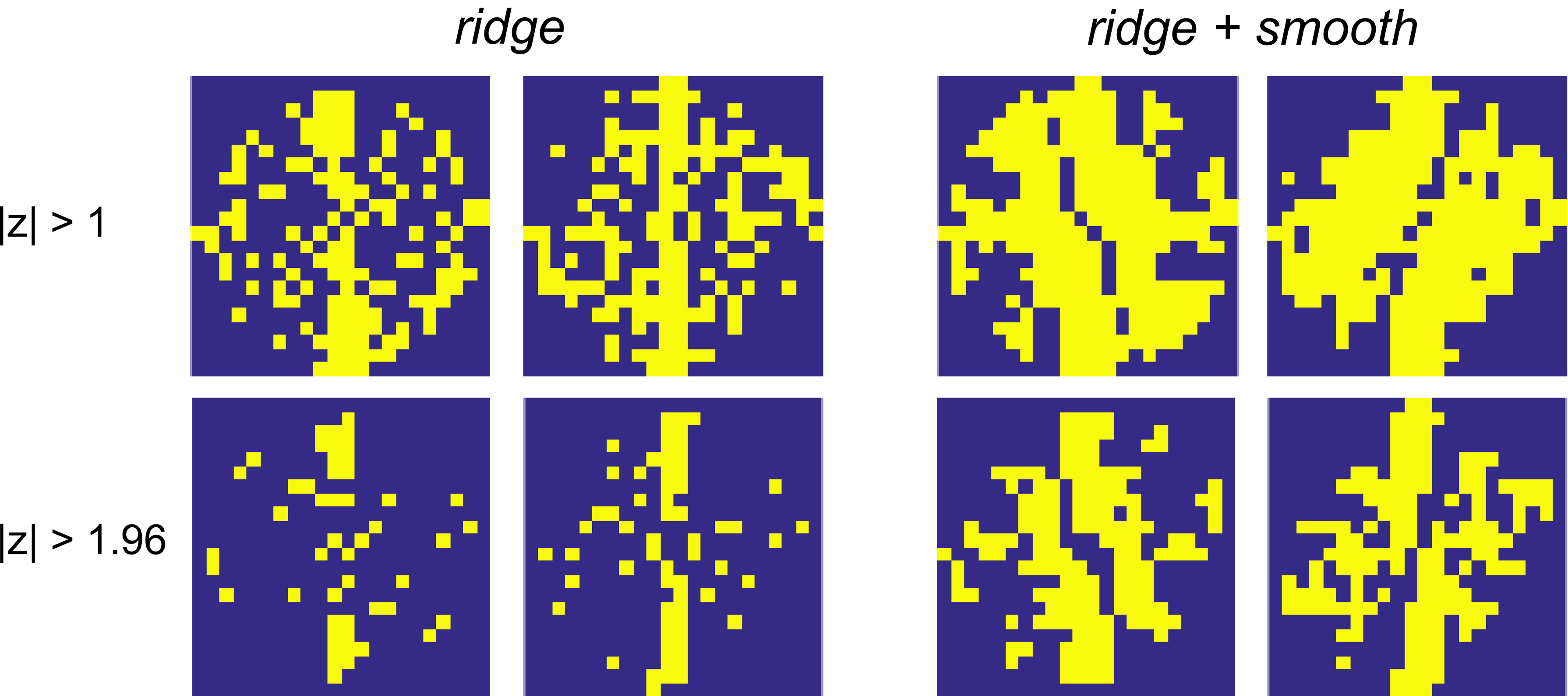

Supplement: S15 Fig — (TIF) [file pcbi.1006829.s015.tif]

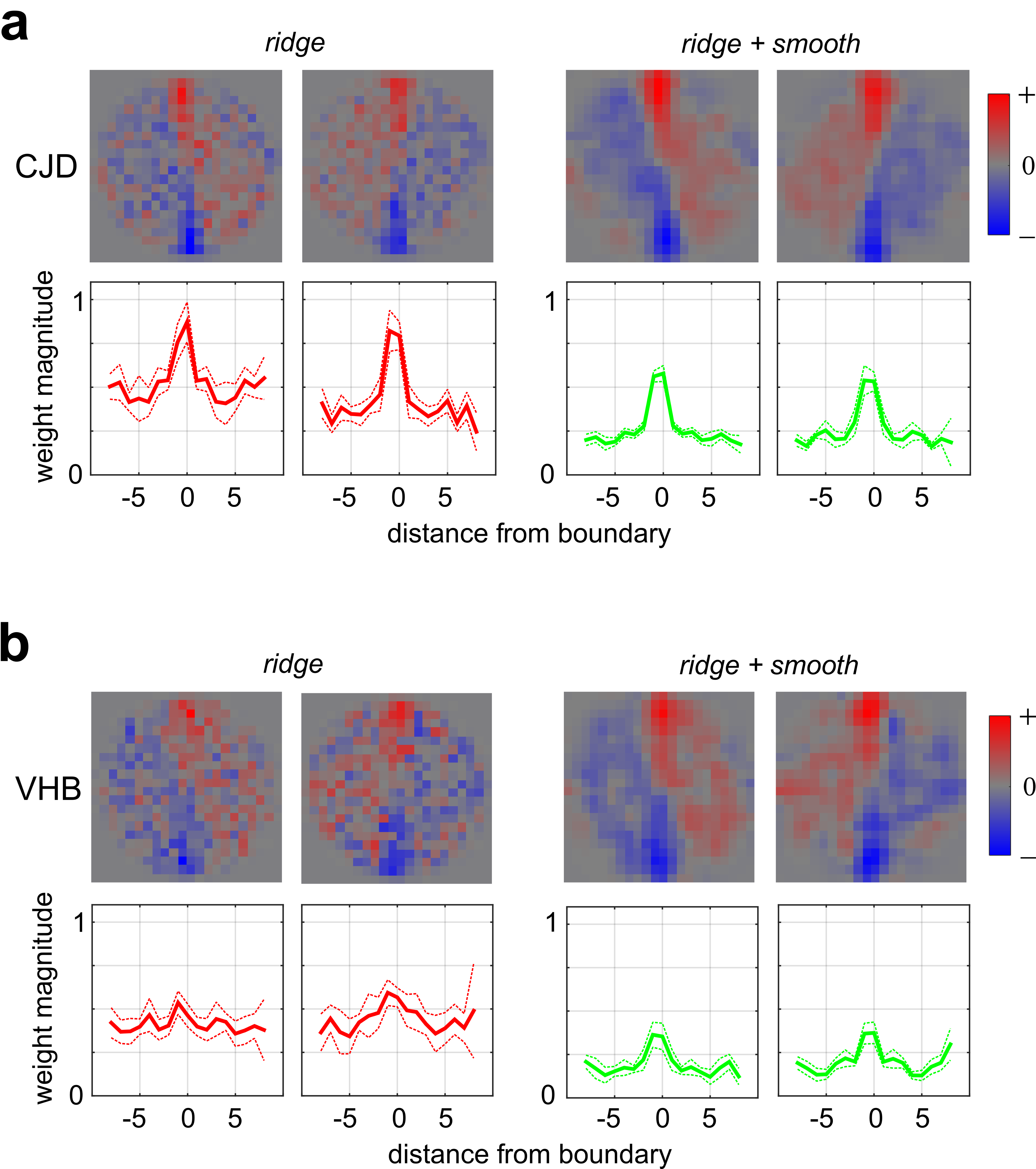

Supplement: S16 Fig — (a) Experiment 2-VAR. (b) Experiment 2-FIX. (TIF) [file pcbi.1006829.s016.tif]

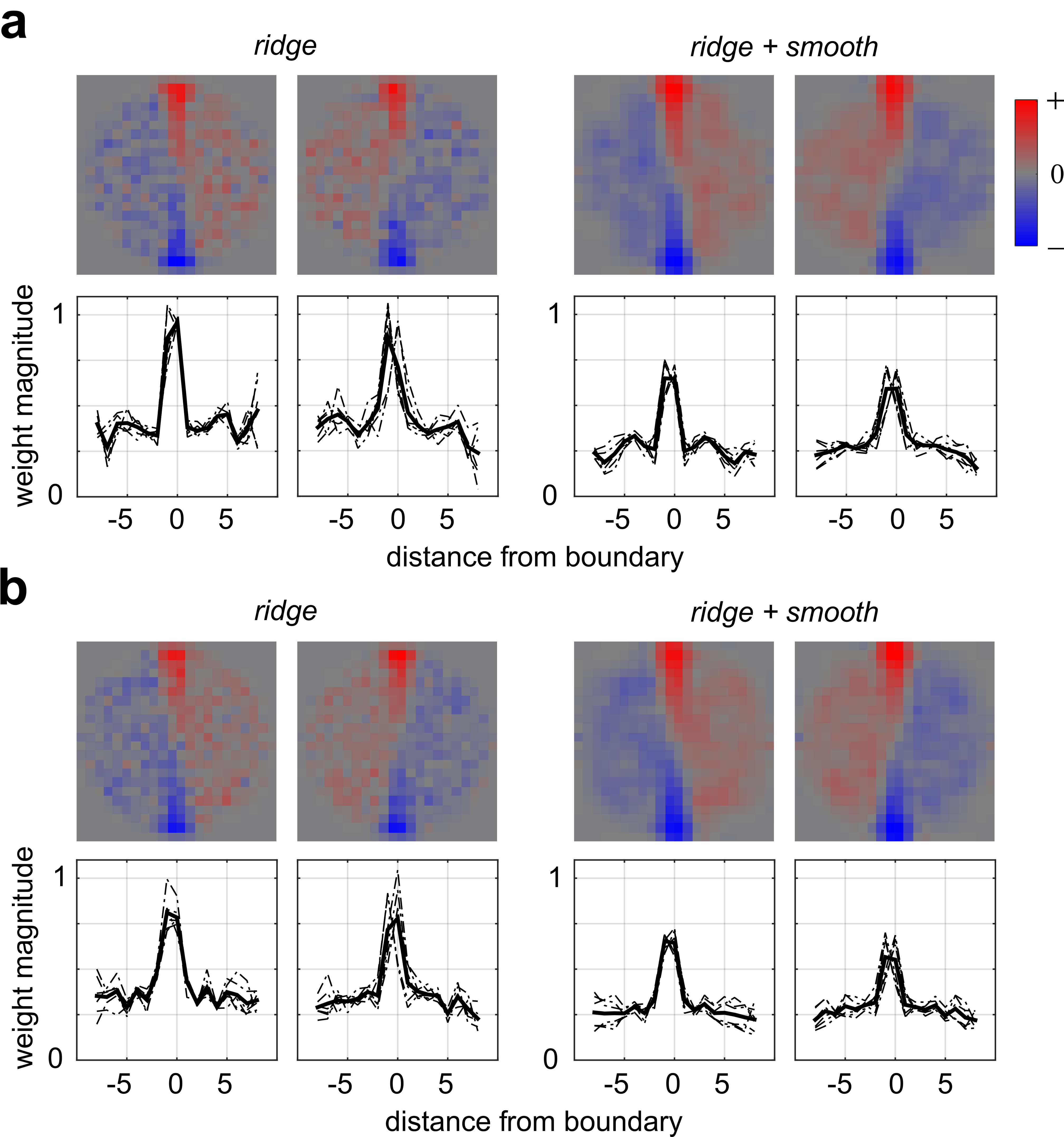

Supplement: S17 Fig — Organization as Fig 3c. (a) Experiment 2-VAR. (b) Experiment 2-FIX. (TIF) [file pcbi.1006829.s017.tif]

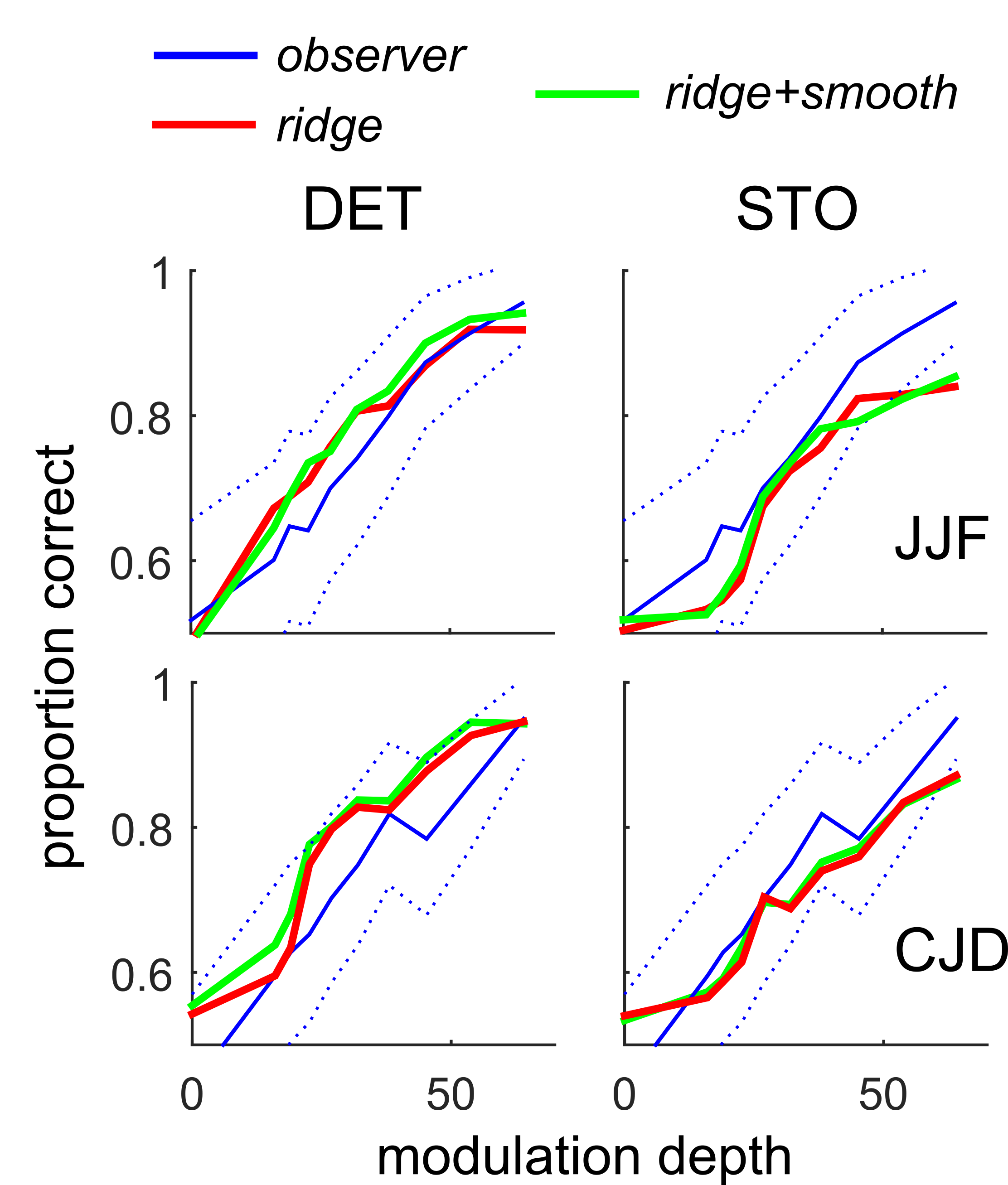

Supplement: S18 Fig — Organization as in Fig 6. (TIF) [file pcbi.1006829.s018.tif]

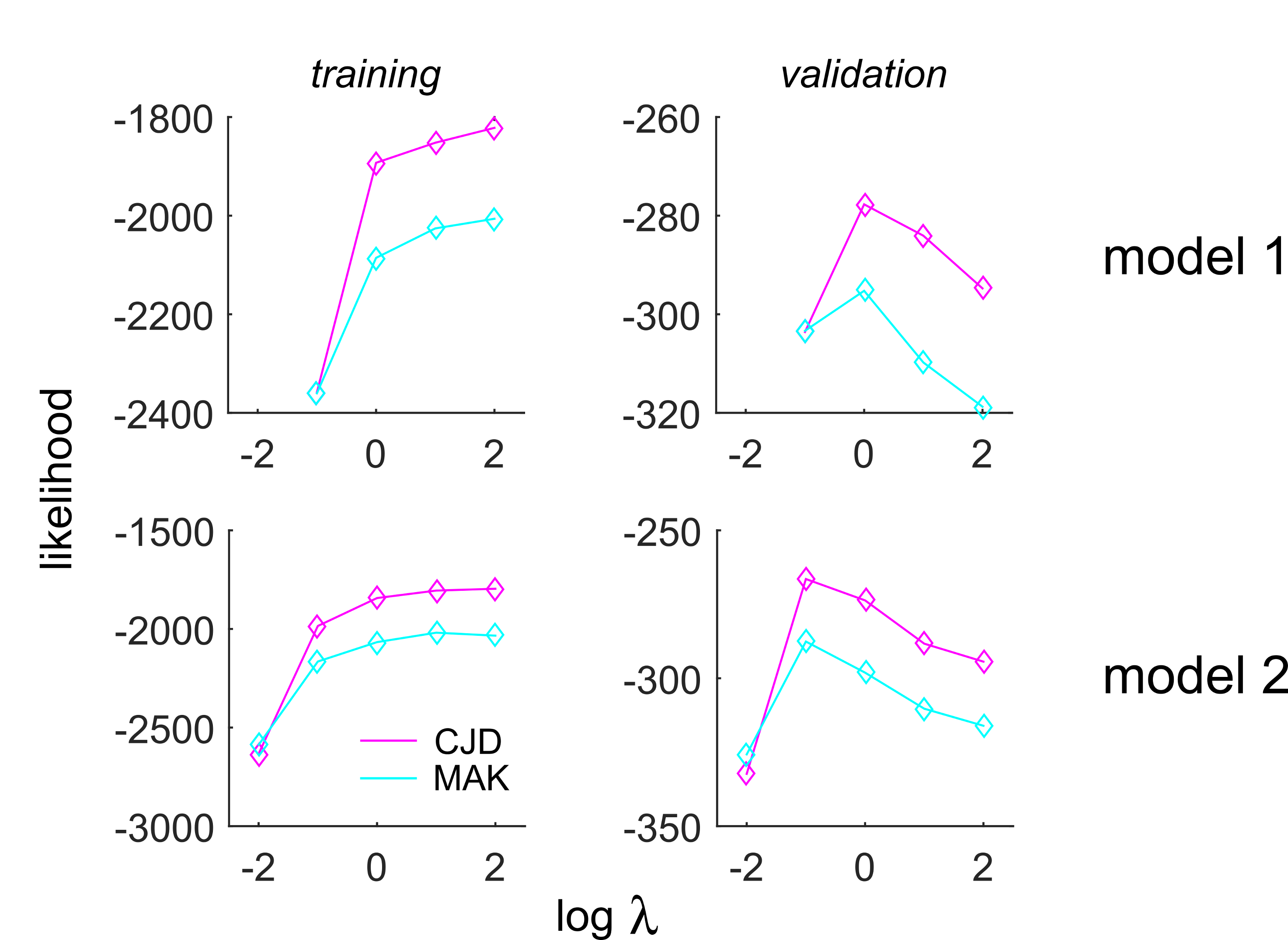

Supplement: S19 Fig — Top: Model 1, Bottom: Model 2. We see that values of the ridge regression hyper-parameter λ in the range 0.1–1 lead to the best generalization for each model. Model 1 generalized best with λ = 1, Model 2 with λ = 0.1. (TIF) [file pcbi.1006829.s019.tif]

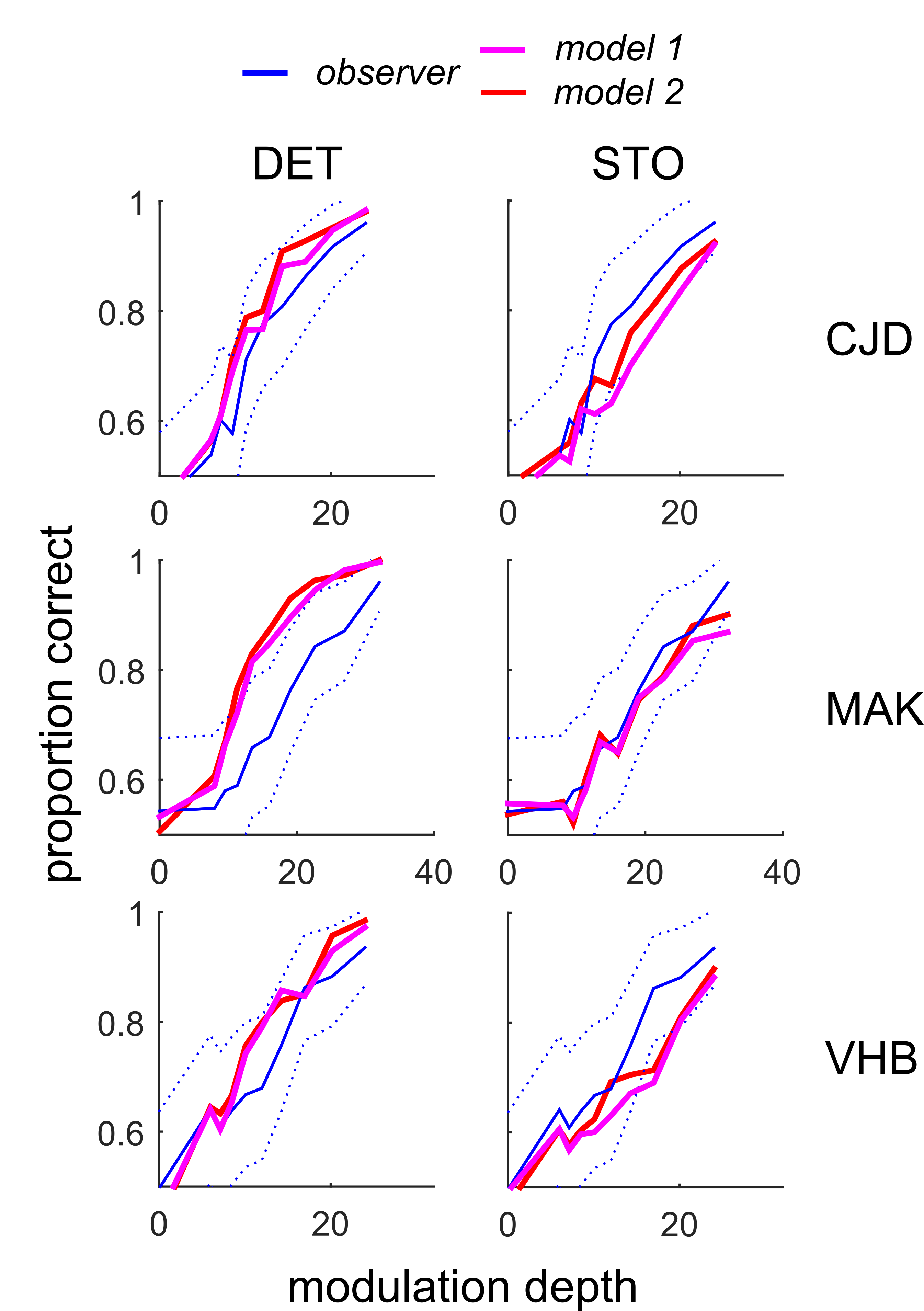

Supplement: S20 Fig — (TIF) [file pcbi.1006829.s020.tif]

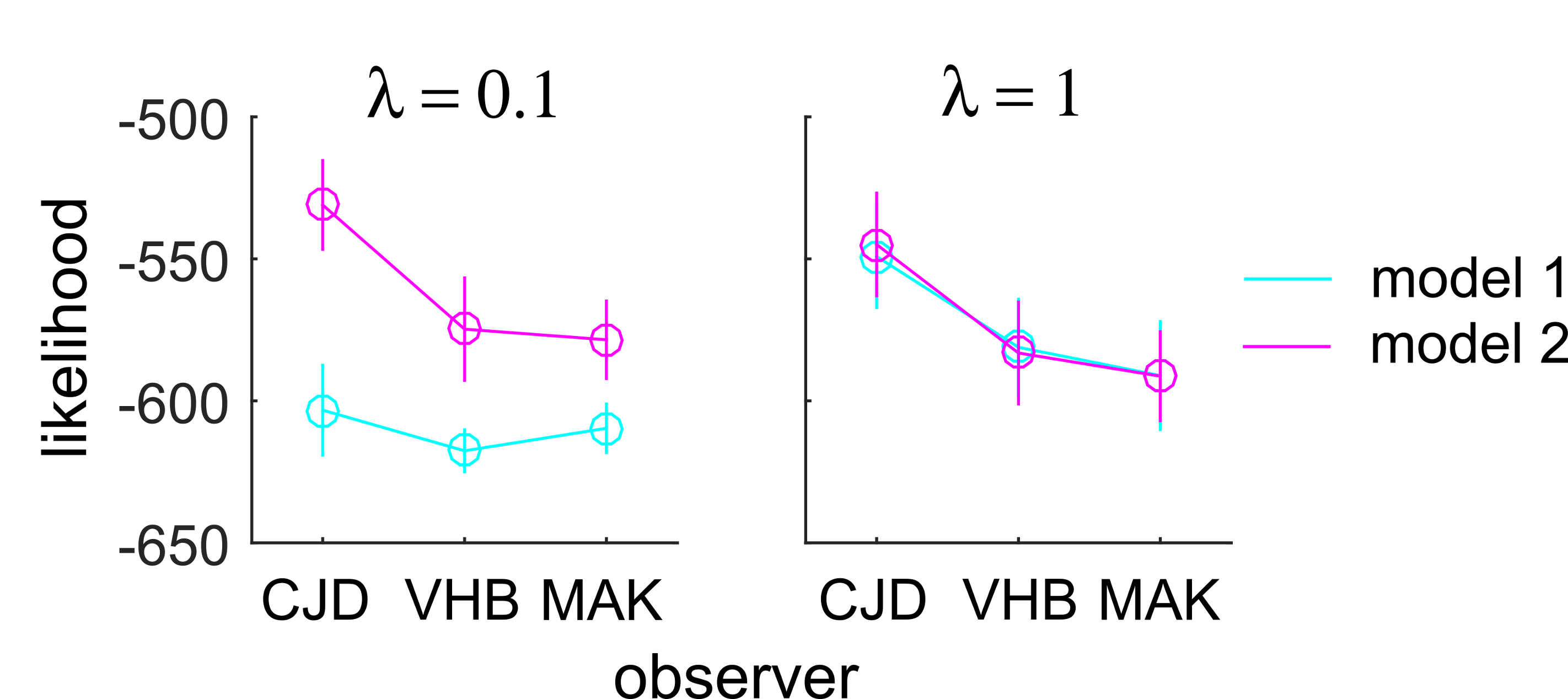

Supplement: S21 Fig — For each model we performed our generalization analysis (Fig 12c) using its optimal value (S19 Fig). (TIF) [file pcbi.1006829.s021.tif]

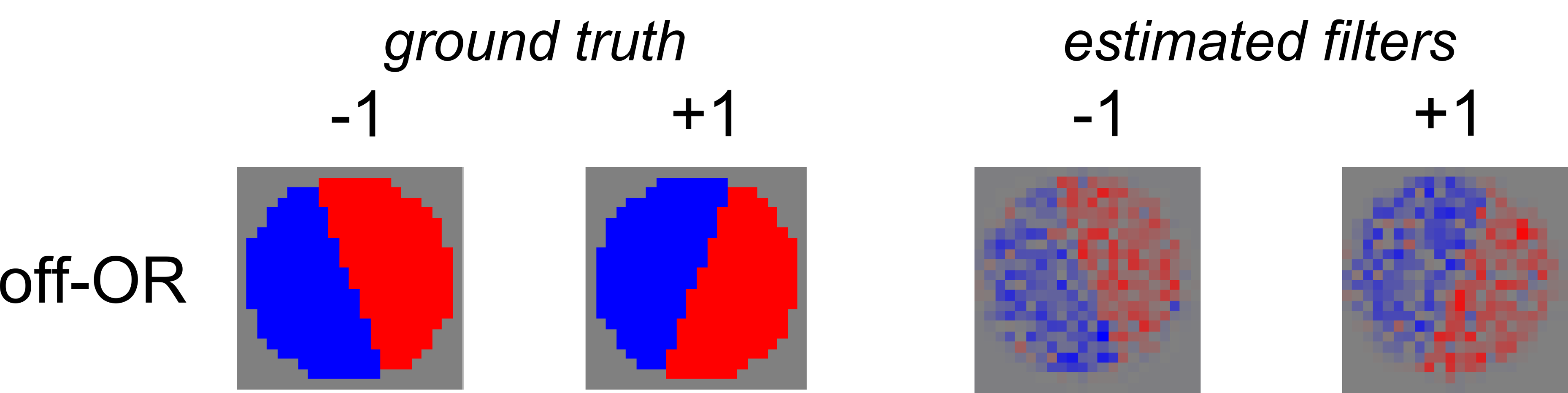

Supplement: S22 Fig — As we see, our method accurately recovers the ground-truth filters, which do not resemble the filters obtained from observers in Experiment 2. (TIF) [file pcbi.1006829.s022.tif]

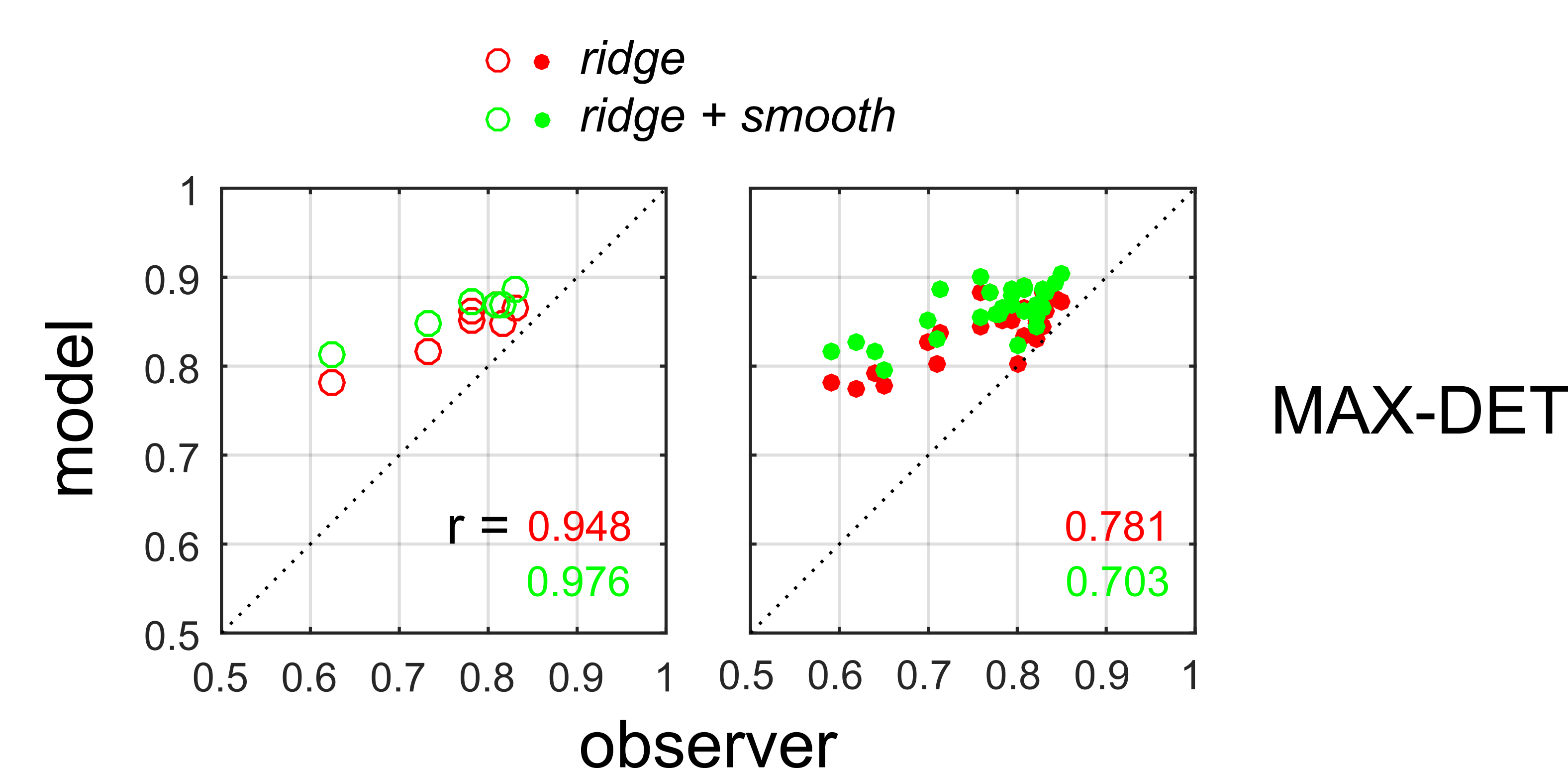

Supplement: S23 Fig — (TIF) [file pcbi.1006829.s023.tif]

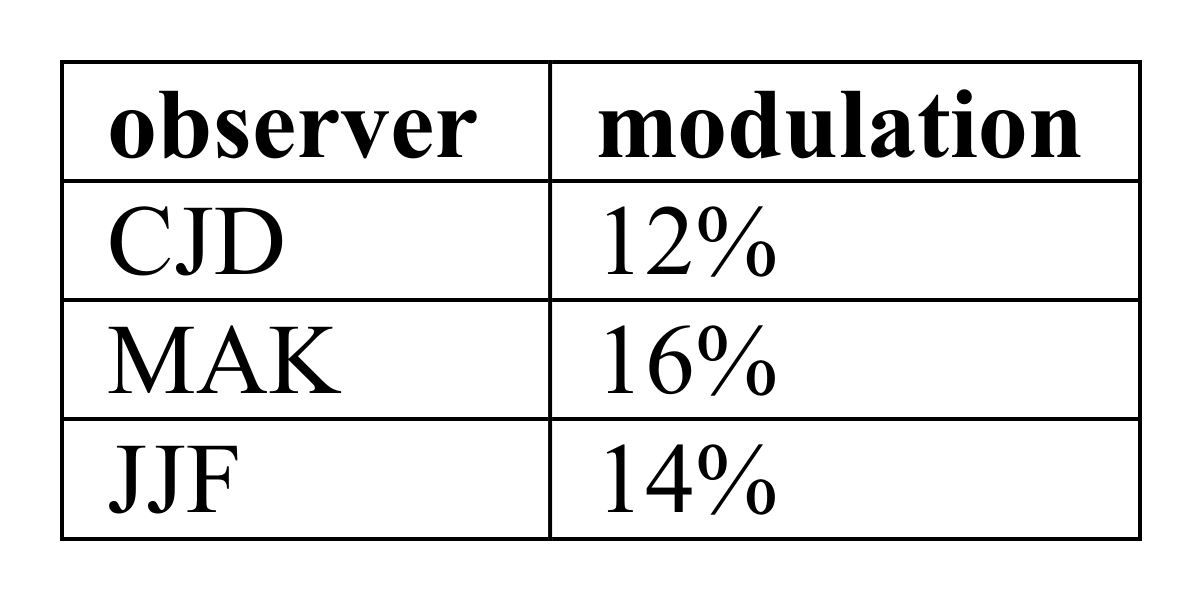

Supplement: S1 Table — Levels were chosen for each subject observer individually to attain near-threshold performance (approximately 80% correct). (TIF) [file pcbi.1006829.s024.tif]

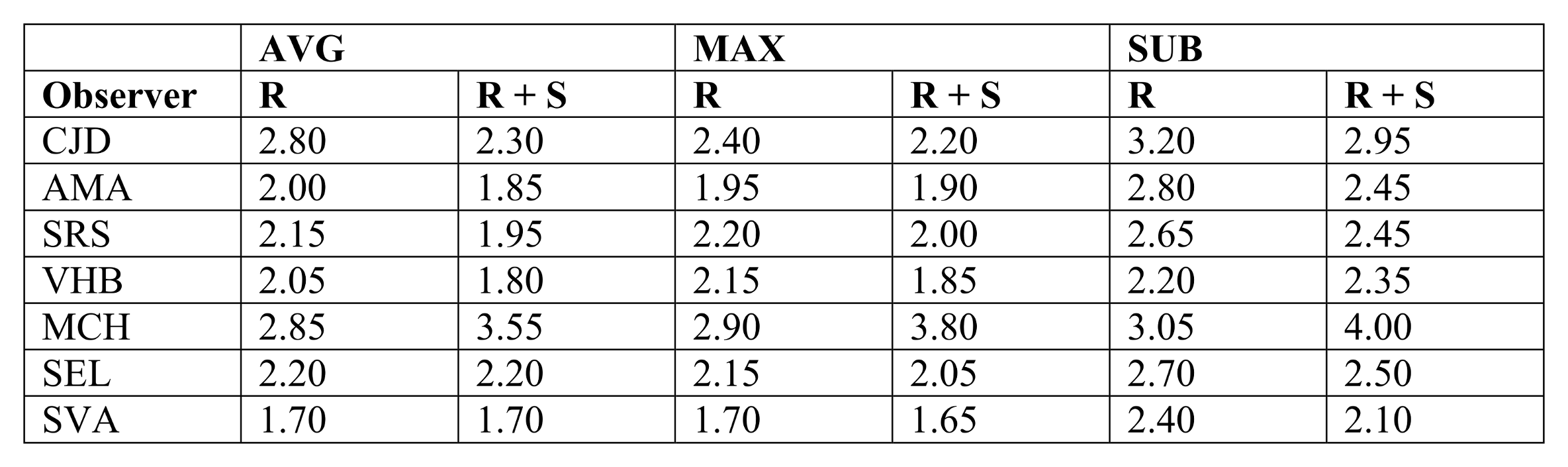

Supplement: S2 Table — (TIF) [file pcbi.1006829.s025.tif]

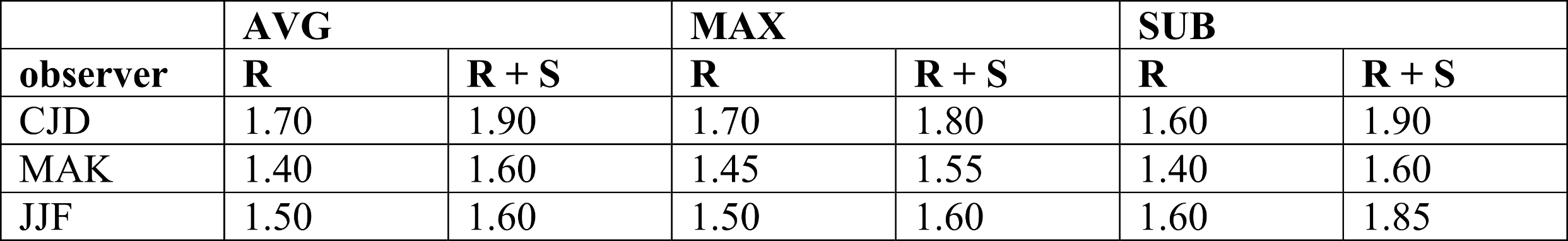

Supplement: S3 Table — (TIF) [file pcbi.1006829.s026.tif]

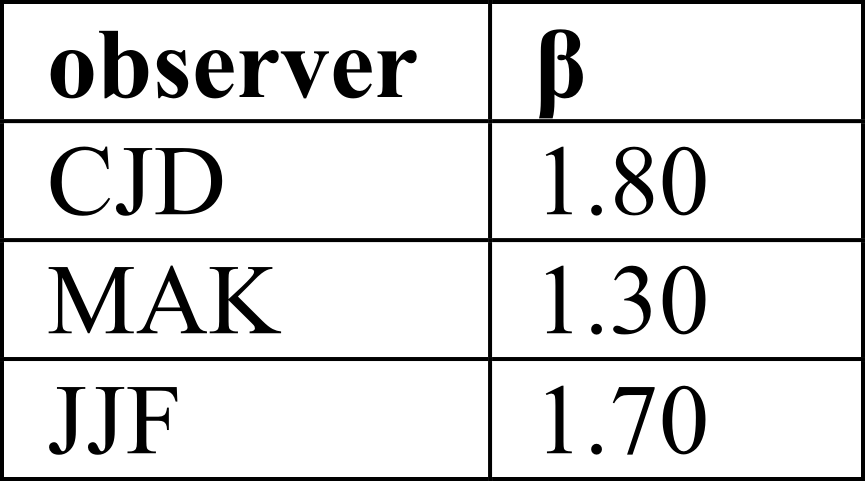

Supplement: S4 Table — Values were optimized on the training set, which was not used for model evaluation. The same value was used for both priors. (TIF) [file pcbi.1006829.s027.tif]

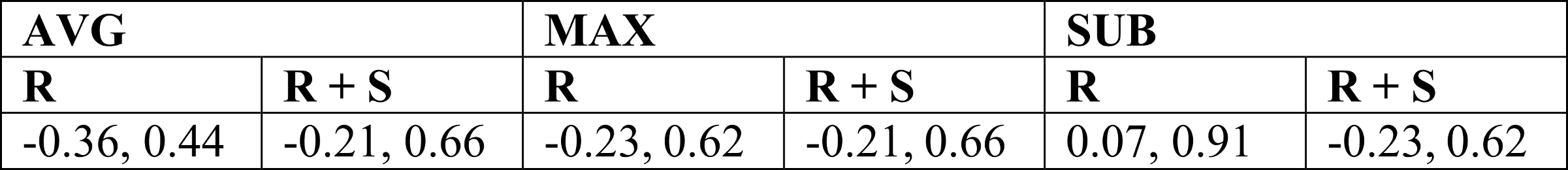

Supplement: S5 Table — (TIF) [file pcbi.1006829.s028.tif]

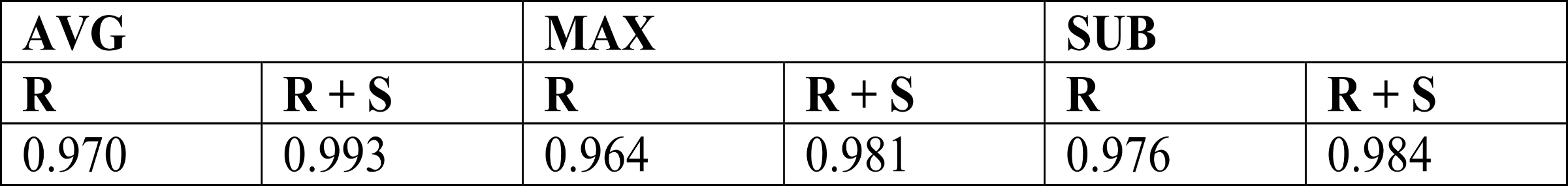

Supplement: S6 Table — For all correlations, p < 0.001. (TIF) [file pcbi.1006829.s029.tif]

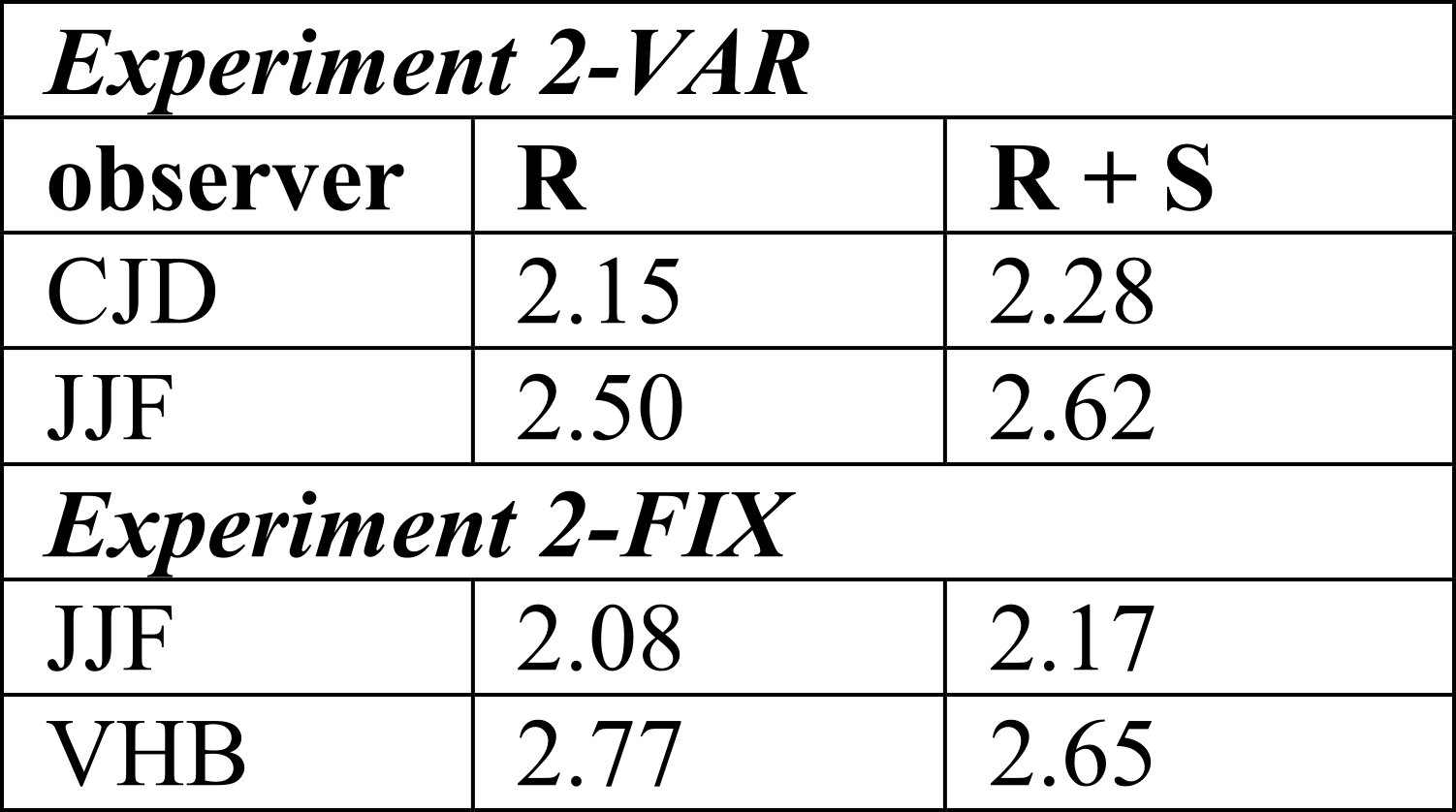

Supplement: S7 Table — (TIF) [file pcbi.1006829.s030.tif]

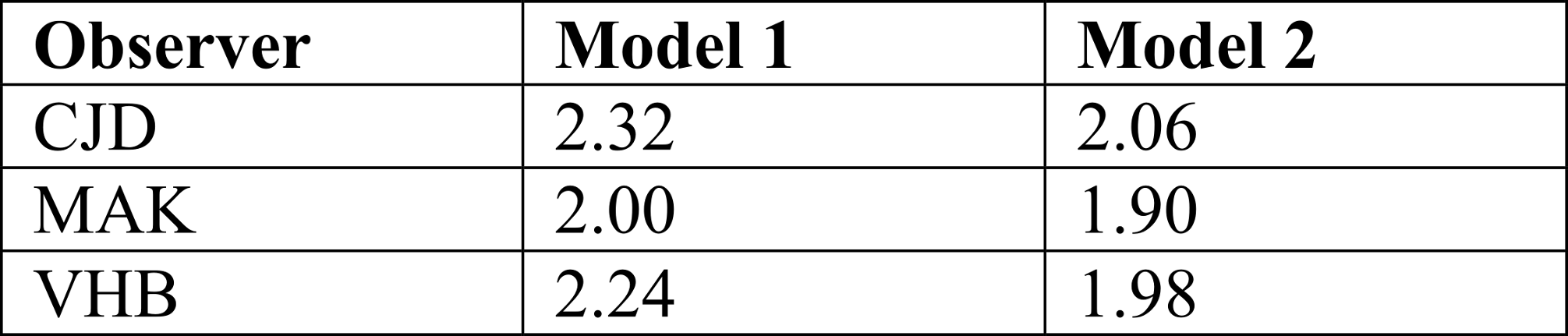

Supplement: S8 Table — Values are obtained by averages over N = 4 training sets. (TIF) [file pcbi.1006829.s031.tif]
